# Synthesis of Flower-Like g-C_3_N_4_/BiOBr and Enhancement of the Activity for the Degradation of Bisphenol A Under Visible Light Irradiation

**DOI:** 10.3389/fchem.2019.00649

**Published:** 2019-10-01

**Authors:** Jun Wu, Yu Xie, Yun Ling, Yunyun Dong, Jian Li, Shiqian Li, Jinsheng Zhao

**Affiliations:** ^1^College of Environment and Chemical Engineering, Nanchang Hangkong University, Nanchang, China; ^2^College of Chemistry and Chemical Engineering, Liaocheng University, Liaocheng, China

**Keywords:** BiOBr, g-C_3_N_4_, photocatalysts, heterojunctions, pollutant degradation

## Abstract

The high recombination rates of photogenerated electron-holes greatly inhibit the catalytic activity of semiconductor photocatalysts. Herein, the heterojunctions of the flower-like g-C_3_N_4_/BiOBr composites were synthesized as photocatalysts by a simple hydrothermal process. The X-ray diffraction, scanning electron microscopy, and X-ray photoelectron spectrometer were utilized to characterize the sample's structure and light absorption properties. The results demonstrated that BiOBr-g-C_3_N_4_-4:1 showed excellent photocatalytic properties and 96.6% of bisphenol (BPA) was removed in 120 min with illumination of visible light due to its narrower band gap than that of pure BiOBr. BiOBr offer little electrons during the photocatalytic reaction. Moreover, the heterostructure between BiOBr and g-C_3_N_4_ facilitates the separation of photogenerated carriers. Excellent stability was exhibited after five cyclic degradation of methyl orange (MO) with the illumination of visible light. The active species trapping experiment indicated that superoxide radical anions (${\text{O}}_{2}^{•-}$) and hole (h^+^) have a great effect on the reaction. A possible mechanism was proposed to explain the whole process of photocatalytic reaction.

**Graphical Abstract:**
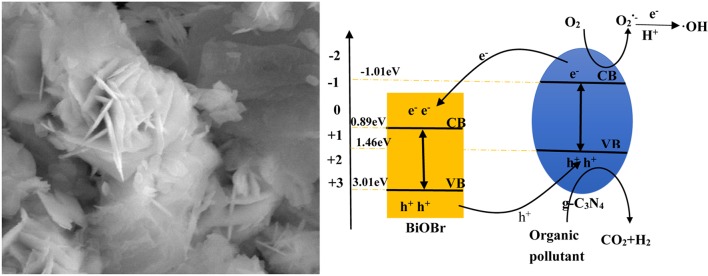
The flower-like BiOBr-g-C3N4 nanosheet and possible mechanism for BPA degradation.

## Highlights

- Flower-like g-C_3_N_4_/BiOBr nanosheets were prepared by a facile hydrothermal method.
- The red-shift of the absorption peak of BiOBr-g-C_3_N_4_ composite was observed.
- The BiOBr-g-C_3_N_4_-4:1 exhibited the highest photocatalytic performance.

## Introduction

Bisphenol A (BPA) is named as 2,2-(4,4-dihydroxydiphenyl) propane, and can be synthesized from phenol and acetone in an acidic medium, which is an important raw material for epoxy resin, polycarbonate, polysulfone, polyarylate, and other products (Tsai, 2006). A primary problem of this material is that upon the release of BPA into the environment, it needs to be degraded in order to become a harmless substance. Several biotic and abiotic methods have been proposed to degrade BPA in wastewater. For example, the ready biodegradability of BPA was evaluated by Athanasios S. Stasinakis by using activated sludge as inoculum with the OECD method 301F (manometric respirometry test) that measures O_2_ production (Stasinakis et al., 2008). In the meantime, a lot of abiotic methods (chemical and physical methods) have been reported. Wu reported BPA degradation by means of releasing nano-TiO_2_ into a water environment with BPA at low concentration (Wu et al., 2016). It was proved that the abiotic degradation rate of BPA was significantly facilitated with the illumination of light. However, at present, both biodegradation and non-biological reduction have the disadvantages of low efficiency and cumbersome. In order to solve the problem, the artificial photocatalytic process furnishes a viable strategy and aroused great interest among researchers (Sun et al., 2017; Liu et al., 2019).

In 1972, the TiO_2_ electrode was employed for the decomposition of water to produce hydrogen and oxygen by Fujishima and Honda (1972), which opened up a new era of researchers looking for semiconductor photocatalysts (Maeda et al., 2006). Nevertheless, on the one hand TiO_2_ just works on the radiation of ultraviolet light because of its wide band gap and ultraviolet light only accounts for ~5% of sunlight (Fu et al., 2012). On the other hand, the large band-gap oxide semiconductor has short exciton diffusion lengths when TiO_2_ is applied in photoelectrochemical devices. Therefore, it is mainly the carriers generated in the space charge layer that contribute to the photocurrent (Santato et al., 2001). A novel semiconductor photocatalyst was investigated to be an alternative to TiO_2_-based materials (Zou et al., 2001; Osterloh, 2008). A polymeric graphitic carbon nitride (g-C_3_N_4_) aroused great interest in the field of semiconductor photocatalysts (Wang and Antonietti, 2012). A metal-free catalyst that contains carbon and nitrogen elements and a small amount of hydrogen, it has a narrow band gap which has a wide visible light absorption rage in the visible region, and high thermal and chemical stability thanks to its tri-s-triazine ring (Wang and Antonietti, 2012; Chen et al., 2014; Zhou et al., 2014). However, pure g-C_3_N_4_ has poor photocatalytic performance regarding limited organic pollutants due to its low quantum efficiency, which limits its practical applications (Liu et al., 2010; Yan et al., 2010). So far, great efforts have been taken to improve the photocatalytic performance of g-C_3_N_4_, such as metal and nonmetal elements doping (Zhang et al., 2009; Liu et al., 2010; Wang et al., 2010), designing different morphology (Groenewolt and Antonietti, 2005; Vinu et al., 2005; Goettmann et al., 2006; Lu et al., 2014), constructing heterostructure with other semiconductor photocatalysts such as ZnO (Kuang et al., 2015), Cu_2_O (Peng et al., 2014), MoS_2_ (Li et al., 2014), Fe_3_O_4_ (Chi et al., 2016), Bi_2_WO_6_ (Wang et al., 2017). Actually, modified g-C_3_N_4_ shows excellent photocatalytic properties because the modification of g-C_3_N_4_ with other photocatalysts promotes the separation of photogenerated carries.

Recently, it was reported that bismuth oxyhalides is a new family of photocatalysts, which showed broad application prospects in the photocatalytic degradation of organic pollutants due to its unique and intrinsic lamellar structures structure as well as the appropriate bandgaps (tetragonal matlockite structure) (Zhang et al., 2006, 2008; Huang et al., 2017a). The structure of the indirect-transition band gap makes the excited electrons emit to the valence band (VB) at a certain K-space distance, thus prolonging the lifetime of the photogenerated carries (Zhang et al., 2006; Chen et al., 2019). In addition, the structures of the [Bi_2_O_2_] plate are interlaced by double slabs of halogen atoms, which provide enough room to polarize the related kernel and orbital. As a resultant, the internal static electrostatic area is perpendicular to the [Bi_2_O_2_] slabs and halogen anionic slabs in BiOX (Zhang et al., 2006; Huang and Zhu, 2008; Ye et al., 2011; Jiang et al., 2012a). Moreover, BiOX exhibited superior practical application in pigments (Maile et al., 2005), catalysts (Kijima et al., 2001), storage materials and ferroelectric materials (Kusainova et al., 2001; Lei et al., 2010). In particular, there is much research on the photocatalytic performance of BiOBr. For instance, Shang's group reported the preparation of a pure BiOBr catalyst with lamellar structure, which was synthesized by hydrothermal method using cetyltrimethylammonium bromide (CTAB) as Bromine source and template and its photocatalytic activity is four times higher than that of its analog made from KBr as the Br source (Shang et al., 2009). Kong's group designed and prepared AgBr-BiOBr heterojunction photocatalysts by varying the loadings of AgBr by an effective co-precipitation method and the obtained catalyst displayed excellent photocatalytic performance on the degradation of Rhodamine B (RhB) under visible-light irradiation (Kong et al., 2012). Thence, there is still a long way to go to find more ways for the preparation of doped BiOBr as photocatalysts.

Herein, we report that a flower-like nanoflake BiOBr-g-C_3_N_4_ with high photocatalytic activity was synthesized by a simple one step hydrothermal method. The CTAB was applied as Br source and template. The catalytic activity of the prepared samples of BiOBr-g-C_3_N_4_ were surveyed by the degradation of BPA in visible light (lambda > 420 nm). A 300W Xe lamp was used as light source with ultraviolet light absorbed by filter. The structure and optical properties were characterized by SEM, TEM, XRD, XPS. The result demonstrated that BiOBr-g-C_3_N_4_ heterojunctions were successfully prepared and a possible mechanism of photocatalytic degradation was proposed. The heterojunction exhibited higher photocatalytic activity than that of pure BiOBr or g-C_3_N_4_. Besides, the flower-like structure was synthesized by controlling experimental condition.

## Experimental

### Photocatalytic Preparation

#### Materials

Melamine was bought form Sinopharm Chemical Reagent Co., Ltd. Ethylene glycol and bismuth nitrate pentahydrate [Bi(NO_3_)_3_·5H_2_O] was purchased from Xilong Chemical Co., Ltd. CTAB was acquired from Shanghai Zhanyun Chemical Co., Ltd. All reagents are analytical pure on this work.

#### Preparation of Bulk g-C_3_N_4_

The bulk g-C_3_N_4_ nanoplates were made according to previous report (Liang and Zhu, 2016). 2 g of melamine was added to the porcelain crucibles with a cover. And then, the crucibles were moved to a Muffle furnace and heated to 550°C for 4 h at a heating rate of 5°C min^−1^. The product is claimed and cooled to room temperature. Collecting the yellow products and grinding it into powder for further use.

#### Preparation of BiOBr-g-C_3_N_4_ Photocatalysts

BiOBr-g-C_3_N_4_ photocatalysts were synthesized by a sample hydrothermal method with different ratio. To begin with, measured a certain amount of CTAB and the as prepared g-C_3_N_4_ were added into 16 ml of ethylene glycol and the mixture was stirred vigorously for 30 min to obtain a uniform suspension at room temperature. Meantime, A certain amount of Bi(NO_3_)_3_·5H_2_O solid was dissolved in 12 ml of nitric acid solution and be stirred for another 30 min to obtain a clear solution. Subsequently, the solution was poured rapidly into the suspension and the mixture was ultrasonicated for another 30 min at room temperature. The obtained mixture was moved to 50 ml Teflon-lined autoclave. Afterward, the autoclave was heated in a constant temperature drying box, and the temperature was kept at 160°C for 12 h. And then, the autoclave was cooled to room temperature after thermal polycondensation. The precipitation was obtained by centrifugation. The precipitation was washed with deionized water and absolute ethanol for three times, respectively. The samples were transferred to oven to dry at 80°C for all night. As a result, different mass ratios of samples were named BiOBr-g-C_3_N_4_-a:b (a:b = 8:1, 4:1, 1:1, 1:4, where a:b was the mass ratios of BiOBr to g-C_3_N_4_, respectively). Otherwise, it was worth noting that the quantity of BiOBr was obtained by measuring raw materials of Bi(NO_3_)_3_·5H_2_O and CTAB.

### Characterization

The X-ray diffraction (XRD) pattern of the samples were measured by RiGdku RINT 2000 X-ray diffractometer (λ = 1.5418Å) with a CuKα radiation source. Scanning electron microscope (SEM) was used for observing the structure of pure BiOBr and BiOBr-g-C_3_N_4_ composites. The optical properties for samples were detected by Spec-3700 DUV Shimadzu UV-visible spectrophotometer. The elemental composition and chemical states were measured by X-ray photo-electron spectroscopy (XPS). The amount of BPA in the aqueous solution was measured by the use of a high-performance liquid chromatograph (HPLC, Agilent 1260), equipped with a photo diode array detector (PDAD) and a ZORBAX Eclipse XDB-C18 column.

### Photocatalytic Activity Test

The catalytic activities of samples were assessed with photocatalytic degradation of Bisphenol A (BPA) under the illumination of visible light. For the visible-light photocatalytic study, a 300W Xe lamp (PLS-SXE300, Beijing, Perfectlight Technology Co., Ltd.) was used as the visible light source with a 420 nm cutoff filter. In each experiment, 10 mg of catalyst power was added into the BPA solution (50 ml, 10 mg L^−1^) by continuous stirring at normal atmospheric temperature. Prior to the irradiation, the suspension was magnetically stirred in dark for 30 min to establish adsorption-desorption equilibrium. In given time intervals, collecting 3 mL of supernatant and the catalyst particles were removed by centrifugation. The content of the BPA in the supernatant was analyzed by HPLC.

### Photocurrent Tests

The separation efficiency of photogenerated electron-hole pairs was recorded on CHI660D electrochemical workstation by using a standard three-electrode system. Saturated sodium sulfate solution was used as electrolyte. The graphite electrode and Ag/AgCl electrode was selected as counter electrode and reference electrode, and the samples derived electrodes were served as the working electrode. A 300W Xe lamp (PLS-SXE300, Perfectlight Company, Beijing, China) of 100 mW/cm^2^ UV-Vis light intensity was employed as light source. For preparing the working electrodes, 10 mg of sample was dispersed in 1 mL of absolute ethanol to obtain suspension, and the suspension was ultrasonicated for 10 min. The slurry was dipped and coated on the surface of fluorine-tin oxide (FTO) glass substrates, which was dried at room temperature overnight.

## Results and Discussion

### Crystal Phase and Morphology Characterization

**Figure 1:**
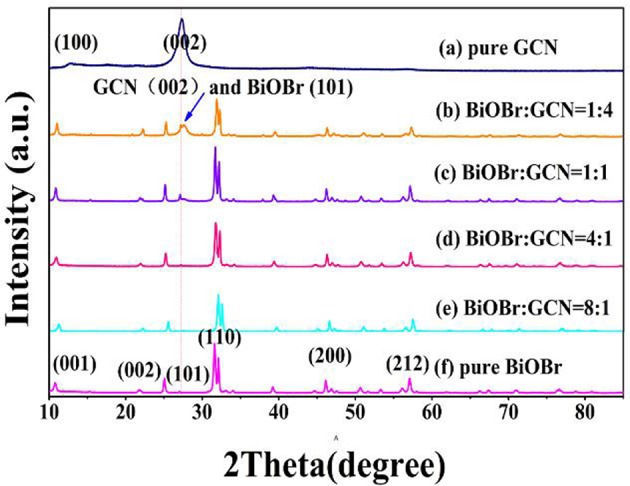
XRD pattern of BiOBr-g-C_3_N_4_ composites, pure BiOBr and g-C_3_N_4_.

Figure 1 represents the X-ray pattern of the BiOBr-g-C_3_N_4_ composites, and the patterns of pure g-C_3_N_4_ and pure BiOBr. The g-C_3_N_4_ exhibits two different diffraction peaks. The high-intensity peak at 26.50° was related to (002) crystal plane of g-C_3_N_4_, attributing to the accumulation of aromatic segments, and the low-peak at 13.028° was related to (100) crystal plane, corresponding to the in-plane structural filling order (JCPDS 87-1526). The result was consistent with previous reports (Yan, 2012; Chang et al., 2013; Hao et al., 2019). The X-ray diffraction peaks of pure BiOBr at 10.907°, 21.914°, 32.241°, 46.241°, and 57.162° were corresponded to (001), (002), (110), (200), and (212) crystal plane of BiOBr, respectively, which can correspond to tetragonal BiOBr (JCPDS 09-0393). All peaks of BiOBr could be found in the patterns of pure BiOBr and BiOBr-g-C_3_N_4_ composites and no other impurity peaks can be observed, which indicates that BiOBr and g-C_3_N_4_ composites were successfully synthesized by a hydrothermal method. The diffraction peak of the (001) plane of BiOBr-g-C_3_N_4_ was shifted to an higher diffraction angle, indicating the interaction between BiOBr and g-C_3_N_4_ contributes to the formation of BiOBr-g-C_3_N_4_ heterojunction (Ye et al., 2013; Yang et al., 2017; Zhang et al., 2019). Otherwise, the sharp diffraction peak of the BiOBr stage display that BiOBr has high crystallinity in this heterojunction. No significant change in the position of the diffraction peak was observed in BiOBr-g-C_3_N_4_ composites, implying that the size and structure of the photocatalysts were not destroyed.

**Figure 2:**
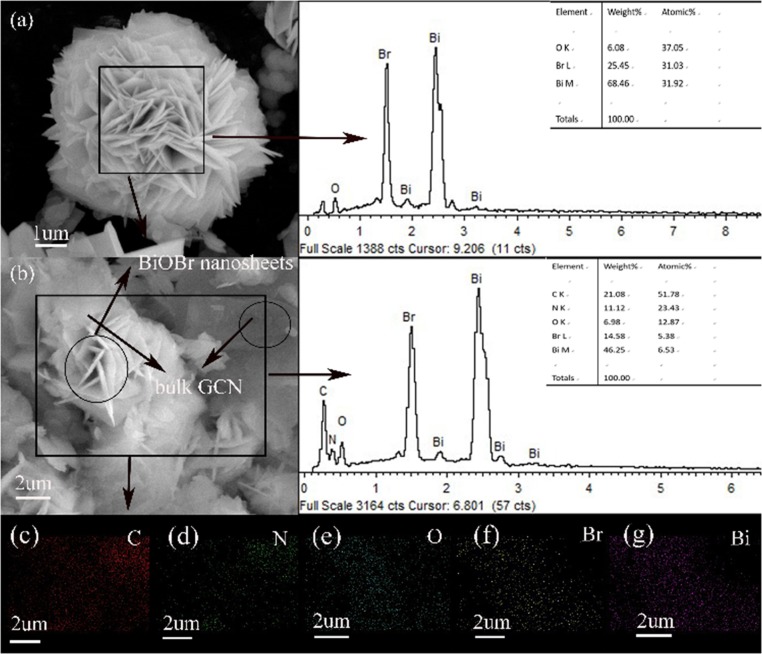
SEM images of **(a)** pure BiOBr and **(b)** BiOBr-g-C_3_N_4_-4:1, elements mapping of BiOBr-g-C_3_N_4_-4:1.

The surface morphology and structure of pure BiOBr and BiOBr-g-C_3_N_4_-4:1 heterojunction was exhibited in Figure 2. As can be seen in Figure 2a, the pure BiOBr exhibit a regular shape that look like flower, and it is self-assemble from many conventional biological sheets with the thickness of about 30–50 nm. Each large nanosheet is formed from a stack of many thin nanosheets. Figure 2b shows the morphology of BiOBr-g-C_3_N_4_-4:1 composite, the g-C_3_N_4_ and BiOBr nanoflakes could be observed. Unlike the pure flower-like BiOBr nanoflake, the nanoflake structure of BiOBr was destroyed, and be interwoven with g-C_3_N_4_ in the BiOBr-g-C_3_N_4_ composite. In other words, the introduction of g-C_3_N_4_ inhibits the assembly of BiOBr. However, it could provide a number of nucleation sites for the reaction because of its abundant surface functional groups of g-C_3_N_4_ (Di et al., 2015). The bulk g-C_3_N_4_ was formed on the surface of BiOBr, which was propitious to form BiOBr-g-C_3_N_4_ heterojunction from the close contact between these two components. The C, N, Bi, O, Br elements were present in BiOBr-g-C_3_N_4_-4:1 composite according to the EDS spectra in Figures 2c–g. Meantime, performing elemental mapping measurements to further determine the presence and distribution of C, N, Bi, O, Br elements, indicating that the BiOBr nanosheets were deposited successfully on the surface of g-C_3_N_4_ and the BiOBr-g-C_3_N_4_ heterojunctions were synthesized successfully. The result is in accordance with the result observed from the XRD patterns.

### Band Gap and Chemical State Analysis

**Figure 3:**
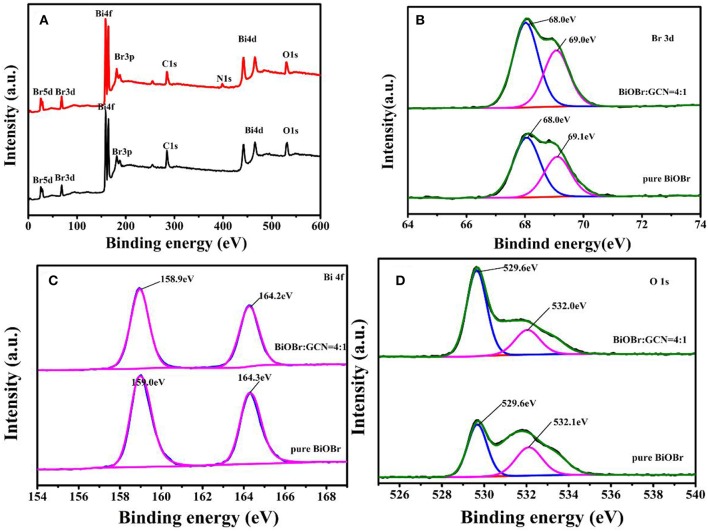
XPS spectra of pure BiOBr and BiOBr-g-C_3_N_4_-4:1 composite; **(A)** survey, **(B)** Br 3d, **(C)** Bi 4f, and **(D)** O1s.

The elemental composition and chemical states were recorded on X-ray photoelectron spectroscopy. The C, N, Bi, O, Br elements were observed according to the XPS survey scan spectrum shown in Figure 3A, further revealing that BiOBr-g-C_3_N_4_ heterojunctions were synthesized successfully, which were consistent with the above-mentioned EDS patterns. The spectrum of Br 3d was shown in Figure 3B, and two typical peaks at about 68.0 and 69.0 eV were observed and attributed to Br3d_5/2_ and Br3d_3/2_ of Br- in BiOBr-g-C_3_N_4_ composites or pure BiOBr (Huo et al., 2012; Liu et al., 2014; Cheng et al., 2011). No obvious movement in the position of the Br3d peak was observed in Figure 3B, indicating that there is no influence on the energy spectrum of Br by coupling with g-C_3_N_4_ (Wang et al., 2015). As is displayed in Figure 3C, the two peaks at 159 and 164.3 eV belonged to Bi 4f_5/2_ and Bi 4f_7/2_, respectively, corresponding to the Bi element in trivalent oxidation state. It is noted that the two peaks of Bi 4f have a slight shift to low binding energy of 158.9 and 164.2 eV, which may be due to the connection between BiOBr and g-C_3_N_4_ (Fu et al., 2012; Huang et al., 2017b). For O 1s spectrum of pure BiOBr and BiOBr-g-C_3_N_4_ composites Figure 3D, the peak with binding energy at 532.1 eV was converted to low binding energy at 532.0 eV. However, no significant effects were observed in the position of the binding energy at 529.6 eV. The peak at 529.6 eV was corresponding to crystal lattice O atoms (Bi-O) and the peak at 532.0 eV was attached into O-H which is derived from surface absorption of photocatalysts (Huo et al., 2012; Zhu et al., 2012; Liu et al., 2014). The analysis result indicated the formation of the BiOBr-g-C_3_N_4_ heterojunction (Zhang et al., 2019).

### Electronic Transfer Path Analysis

**Figure 4:**
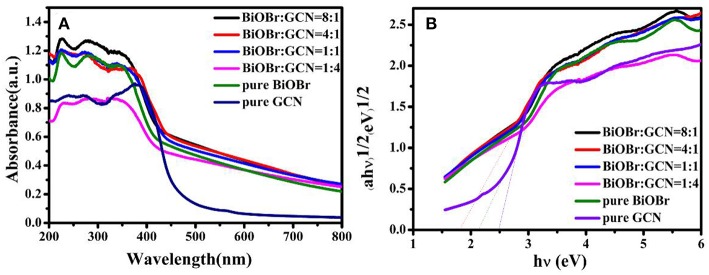
**(A)** UV-vis diffuse reflectance spectra and **(B)** the band gap energies (*E*_g_) of pure BiOBr and g-C_3_N_4_, BiOBr-g-C_3_N_4_ composites.

The electronic transfer path of pure BiOBr, pure g-C_3_N_4_, and BiOBr-g-C_3_N_4_ composites were measured with UV-vis diffuse reflectance spectra (DRS). The fundamental absorption edges of BiOBr and g-C_3_N_4_ at 464 nm and 437 nm were shown in Figure 4, which was consistent with the previous reports (Yan et al., 2009; Jiang et al., 2012b; Liao et al., 2012). The absorption edge of BiOBr-g-C_3_N_4_-4:1 is shifted to longer wavelength (at about 519 nm) than pure BiOBr and g-C_3_N_4_, due to the interaction between BiOBr and g-C_3_N_4_ (Wang et al., 2019). Moreover, the BiOBr-g-C_3_N_4_-4:1 photocatalyst possessed a strong absorption almost in the entire visible-light region. Herein, lots of electrons were excited to form free electrons and moved to the surface of catalyst. Furthermore, the band gap energies of photocatalysts were calculated by follow Kubelka-Munk formula (Luo et al., 2008; Cao et al., 2012):

(1) $$\begin{array}{r} \alpha h\nu =\left(h\nu -{\text{E}}_{\text{g}}\right)\text{n} \end{array}$$

Where, α, ν, h, *E*_g_ means the absorption coefficient, light frequency, Planck's constant and band gap of semiconductors, respectively. In addition, the number (n) is depended on type of semiconductor (*n* = 2 for an indirect transition and *n* = 1/2 for a direct transition). For pure BiOBr and g-C_3_N_4_, the n is equal to 2. The band-gap energies of BiOBr, g-C_3_N_4_, and BiOBr-g-C_3_N_4_-4:1 photocatalysts were separately calculated to be 2.13, 2.47, and 1.80 eV, respectively. As a result, the low band gap is beneficial for the electrons transition from valence band to conduction band. The band edges of photocatalysts were estimated by using the following Equations (Chen et al., 2018):

(2) $$\begin{array}{r} {\text{E}}_{\text{VB}}=\text{X}-{\text{E}}_{0}+0.5{\text{E}}_{\text{g}} \end{array}$$

(3) $$\begin{array}{r} {\text{E}}_{\text{CB}}={\text{E}}_{\text{VB}}-{\text{E}}_{\text{g}} \end{array}$$

**Scheme 1:**
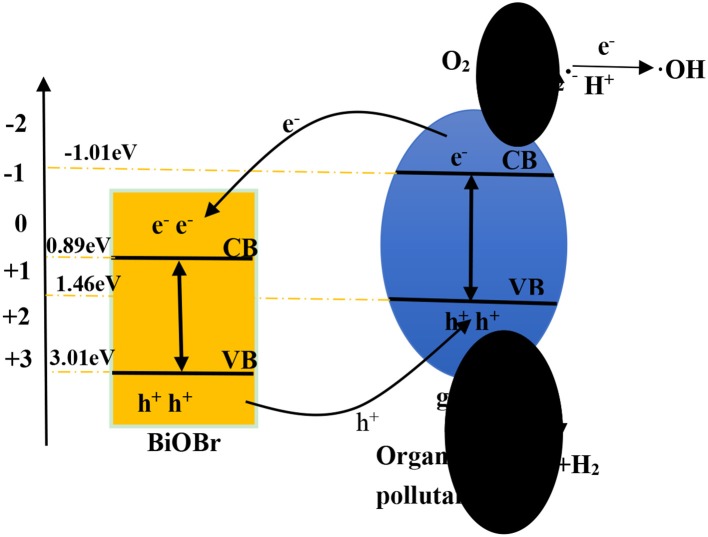
Photocatalytic mechanism scheme under visible-light irradiation.

The X stand for electronegativity of a semiconductor. The value for g-C_3_N_4_ and BiOBr are separately 4.72 and 6.45 eV, respectively. *E*_0_ represents free electrons energy on the hydrogen scale (4.5 eV). Therefore, the valence band edge of BiOBr (+3.02 eV) is more positive than the valence band edge of g-C_3_N_4_ (+1.46 eV). Meantime, the conduction band edge of BiOBr (+0.89 eV) is more positive than the conduction band edge of g-C_3_N_4_ (−1.01eV). The electrons and holes were generated when electrons of valence band were excited under visible light irradiation and migrated from valence band to conduction band. Photogenerated holes on the VB of BiOBr were migrated to VB of g-C_3_N_4_ and photogenerated electrons on CB of g-C_3_N_4_ were migrated to CB of BiOBr. The formation of heterojunction promotes the separation of photogenerated electrons and holes and the redox reaction on the surface was efficient under visible-light irradiation (Scheme 1).

**Figure 5:**
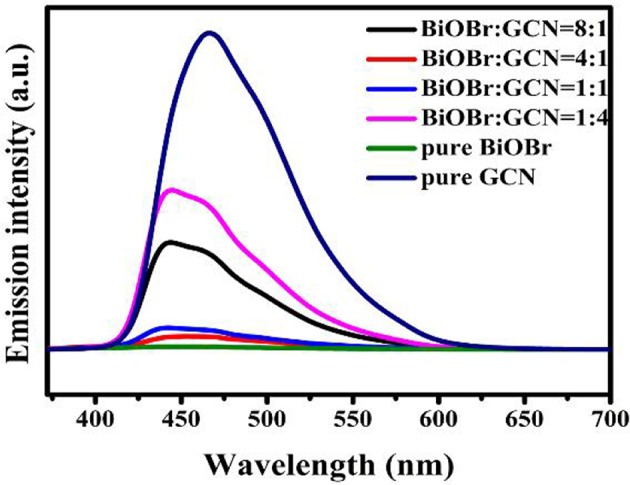
The PL spectra of pure BiOBr and g-C_3_N_4_, BiOBr-g-C_3_N_4_ composites.

Photoluminescence (PL) spectra were utilized to analyze the separation efficiency and transportation of semiconductor photocatalysts. The electrons of the valence band were excited after light irradiation and transferred from VB to CB. However, rapid recombination occurred between electrons and holes due to the gravitational forces of between them, which is just like the force of gravity between man and earth. The PL emission peaks of BiOBr, g-C_3_N_4_, and composites were exhibited in Figure 5. The g-C_3_N_4_ exhibits a strongest emission peak at 466 nm. The blue shift occurred and the peak intensity decreased with the introduction of BiOBr to the heterojunction, and the BiOBr-g-C_3_N_4_-4:1 displayed the lowest emission peak intensity (Figure 5). Considering the fact that BiOBr-g-C_3_N_4_-8:1 even had a higher PL intensity than that of the BiOBr-g-C_3_N_4_-4:1, indicating that the low PL intensities of the BiOBr-g-C_3_N_4_ composites did not originate from the lower amounts of g-C_3_N_4_, but originated from the formation of the heterojunctions, which suppressed the recombination of the photo-generated charges. The band structure of two components in the composite matched well, which impeded the rapid recombination of photogenerated carries within the semiconductors. The lower emission peak intensity, the less possibility in the recombination of photogenerated carries. Moreover, there is not any observed PL signal for the pure BiOBr, indicating that no radiative recombination of photo-generated charges occurred for the pure BiOBr (Sun et al., 2014; Huang et al., 2015a). In summary, the BiOBr-g-C_3_N_4_ heterojunctions were synthesized successfully, and BiOBr provides little electrons separately. The electrons mainly originated from g-C_3_N_4_ and flow from the g-C_3_N_4_ to the surface of BiOBr. The tight contact between BiOBr and g-C_3_N_4_ offers a convenient access, which increases the transfer rate of photogenerated electrons.

**Figure 6:**
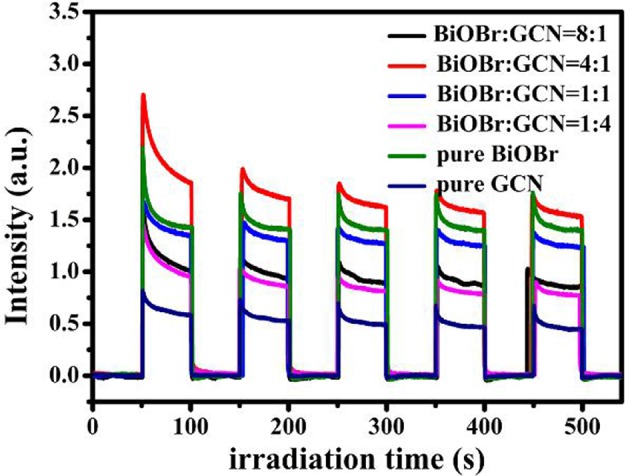
Transient photocurrent of pure BiOBr and g-C_3_N_4_, BiOBr-g-C_3_N_4_ composites.

For offering further evidence to support the enhanced separation efficiency of photogenerated carries, the transient photocurrent-time curves of pure BiOBr and g-C_3_N_4_ were shown in Figure 6, together with BiOBr-g-C_3_N_4_ composites. The higher the photocurrent intensity, the better the photocatalytic activity is. It is the common understanding on the relationship between photocurrent and photocatalytic activity. As shown in Figure 6, the current reached rapidly to the stable state when on the light irradiation was turned on and decreased suddenly after the light turning off. The photocurrent can be maintained to a large extent of the original value after five cycles of the exposure to the on-off switchable irradiation.

BiOBr-g-C_3_N_4_-4:1 exhibited the highest intensity of photocurrent among the samples, indicating that the heterostructure in BiOBr-g-C_3_N_4_ composite greatly inhibited the recombination of photogenerated carries and extends the lifetime of photoinduced electrons (Huang et al., 2015b, 2016).

### Photocatalytic Studies

**Figure 7:**
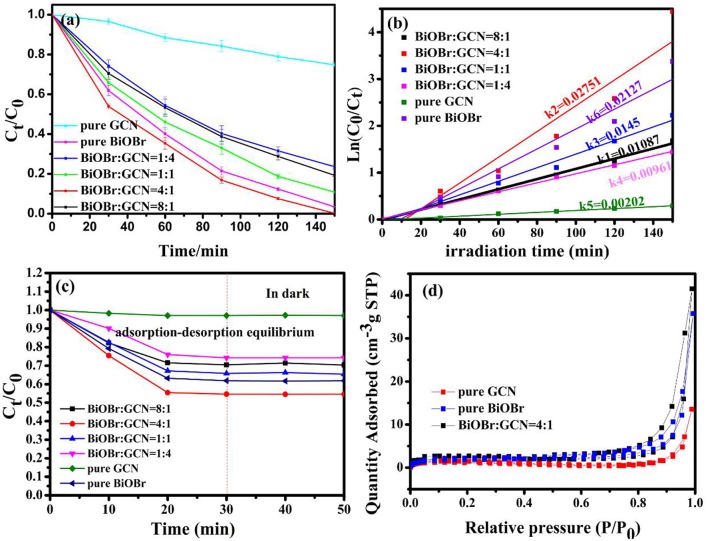
**(a)** Photocatalytic activity; **(b)** plot of In(C_0_/C_t_) vs. irradiation time over pure BiOBr and pure g-C_3_N_4_, BiOBr-g-C_3_N_4_ composites; **(c)** adsorption curves of BPA in the dark; **(d)** N_2_ adsorption-desorption isotherms curves.

The photocatalytic degradation of BPA was utilized to test the degradation efficiency of samples with the cutting off of ultraviolet light (λ < 420 nm). In order to reaching adsorption-desorption equilibrium, the catalyst/BPA solution mixture was stirred for 30 min in the dark. The photocatalytic efficient and error bar were shown in Figure 7a, the BiOBr-g-C_3_N_4_-4:1 material exhibited higher photocatalytic performance than other catalysts prepared previously. The BPA was completely removed within 120 min under visible-light irradiation for the composite catalyst. However, for pure BiOBr and g-C_3_N_4_, 96.6 and 25.2% of BPA were removed, respectively, during the same incubation time. To further investigate the adsorption performance in the dark condition, the catalysts was added into BPA solution as the light was turned off. As was shown in Figure 7c, it can be found that BiOBr/g-C_3_N_4_-4:1 showed the best adsorption capacity, and it showed that almost 50% of BPA was adsorbed in 30 min. The phenomenon was consistent with result of N_2_ adsorption-desorption isotherms curves in Figure 7d. The BET surface area and pore volume of samples were measured, and the result was shown in Figure 7d and Table 1. For three catalysts, the isotherms curves exhibited a type IV curve and type H3 hysteresis loops at the relative pressure of 0.5–1.0. The following pseudo-first-order kinetics equation was used to compare the degradation efficiencies of samples (Fu et al., 2018; Zeng et al., 2018):

(4) $$\begin{array}{r} \text{In}\left({\text{C}}_{0}/C_{\text{t}}\right)={\text{k}}^{*}\text{t} \end{array}$$

Where, C_0_ and C_t_ are the BPA concentrations in solution at time of 0 and t, respectively. The k represents the first-order kinetics rate constant. The result was shown in Figure 7b, which was consistent with above analysis. The BiOBr-g-C_3_N_4_-4:1 exhibited the maximum rate constant of 0.02751 min^−1^, being 1.29 and 13.6 times of pure BiOBr and g-C_3_N_4_ photocatalysts, respectively.

**Table 1:** The parameters obtained from the nitrogen adsorption/desorption of composite.

**Sample**	**BET surface/m^**2**^g^**−1**^**	**Pore volume/cm**	**Band gap/eV**
Pure BiOBr	5.9906	0.0551	2.13
Pure GCN	2.8643	0.0209	2.47
BiOBr/g-C_3_N_4_-4:1	6.0396	0.0642	1.80

**Figure 8:**
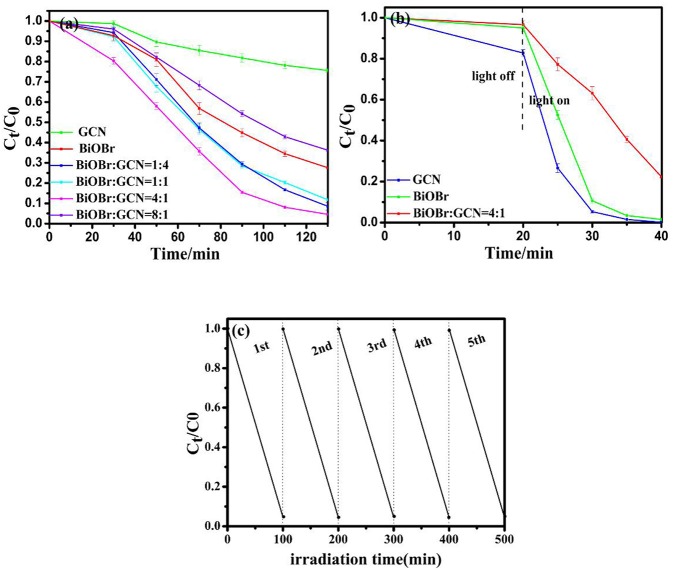
Degradation of **(a)** MO and **(b)** RhB over pure BiOBr, g-C_3_N_4_ and BiOBr-g-C_3_N_4_ composites; **(c)** Recycling tests of BiOBr-g-C_3_N_4_-4:1 photocatalyst on the degradation of MO.

Photocatalytic degradation of methyl orange (MO) and rhodamine B (RhB) over BiOBr, g-C_3_N_4_, and BiOBr-g-C_3_N_4_ composites were applied to further analyze photocatalytic efficiencies of degrading organic pollutants under visible-light irradiation. As shown in Figure 8, the BiOBr-g-C_3_N_4_-4:1 still possessed the highest photocatalytic activity among all samples prepared. 95.5% of MO was decolored in 100 min and 99.9% of RhB was decolored in 20 min over BiOBr-g-C_3_N_4_-4:1 composite. The result demonstrated that BiOBr-g-C_3_N_4_ possessed the ability of degrading organic pollutants in wastewater under visible-light irradiation, with BiOBr-g-C_3_N_4_-4:1 possessed the highest photocatalytic activity. The stability of BiOBr-g-C_3_N_4_-4:1 photocatalyst was further investigated by repeated degradation of MO under visible-light irradiation. The photocatalytic activity showed a slight decrease after five cycles. It was proved that BiOBr-g-C_3_N_4_-4:1 exhibited excellent stability.

### Photocatalytic Mechanism

**Figure 9:**
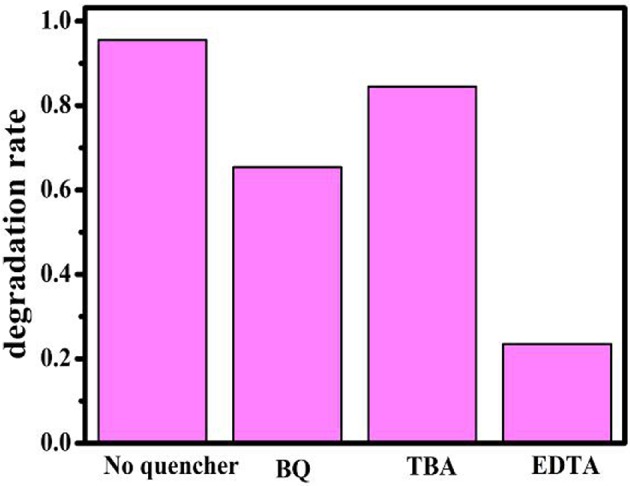
Trapping experiment of active species under visible-light irradiation.

As we all know, active species, such as ${\text{O}}_{2}^{•-}$, h^+^, and •OH radical, according to previous reports, play an important role during the photocatalytic degradation process (Fu et al., 2005; Yang et al., 2015; Huang et al., 2017c). Herein, the active species trap experiment over the BiOBr-g-C_3_N_4_ photocatalyst was carried out with the illumination of visible light. The results of photocatalytic degradation with addition of ethylenediaminetetraacetate (EDTA, a quencher of h^+^), benzoquinone(BQ, a quencher of ${\text{O}}_{2}^{•-}$), and tert-butyl alcohol(TBA, a quencher of •OH) were shown in Figure 9. Degradation of MO was used to achieve this process. For comparison, the experiment was carried out without addition of any scavenger. It was obviously found that photocatalytic efficiency of MO decrease when EDTA and BQ were added into the solution. It turns out that h^+^ and ${\text{O}}_{2}^{•-}$ was the main active radical in the photocatalytic reaction of MO.

According to the above calculated band-gap edge, a possible photocatalytic mechanism was proposed to explain the process of photocatalytic degradation reaction. The conduction band (CB) potential of g-C_3_N_4_ (−1.01 eV vs. NHE) is negative than the standard redox potential of O_2_/${\text{O}}_{2}^{•-}$(−0.046 eV vs. NHE) (Cui et al., 2012). So, free radical ${\text{O}}_{2}^{•-}$ were produced from adsorbed oxygen molecule due to reduction of the electrons in the CB of g-C_3_N_4_. However, the standard redox potential of Bi^4+^/Bi^3+^ (1.59 eV) is negative than *E*_0_ (•OH/OH^−^ = +1.99 eV vs. NHE) (Fu et al., 2005). It can be concluded that •OH could not be obtained on the VB of BiOBr and g-C_3_N_4_. Actually, O_2_ dissolved into water might be reduced to •OH by a two-electron oxidation pathway (Fu et al., 2005; Kanagaraj and Thiripuranthagan, 2017). As a result, the BiOBr-g-C_3_N_4_-4:1 exhibited higher photocatalytic performance than pure BiOBr and g-C_3_N_4_. Meantime, the band gap of BiOBr-g-C_3_N_4_-4:1 is lower than pure photocatalysts. It can be deduced that electrons in the CB of g-C_3_N_4_ were transferred to the CB of BiOBr, which was consistent with PL result.

## Conclusion

In summary, we have reported a simple hydrothermal method to synthesize successfully flower-like BiOBr and BiOBr-g-C_3_N_4_ composites. The results displayed that g-C_3_N_4_ were deposited on the surface of BiOBr nanosheets to form a tight contactheterostructure between BiOBr and g-C_3_N_4_ nanoflakes. The band gap of BiOBr-g-C_3_N_4_ heterojunction was narrower than the band gap of pure BiOBr nanosheet bulk g-C_3_N_4_, which was propitious to the transfer of photogenerated electrons from valence band to conduction band. Moreover, the BiOBr-g-C_3_N_4_-4:1 exhibited the highest photocatalytic performance for the degradation of BPA under visible light irradiation due to its superior quantum efficiency. The PL results demonstrated that the charges were offered mainly by g-C_3_N_4_ and moved from CB of g-C_3_N_4_ to CB of BiOBr. The band edges of the composites were shifted to longer wavelength than pure BiOBr with the loading of g-C_3_N_4_. In order to evaluate the degradation performance of organic pollutants in wastewater, degradation of MO and RhB under visible light irradiation were also carried out at room temperature. The result demonstrated that the composite photocatalyst possessed excellent photocatalytic performance in the degradation of organic pollutants. Last but not least, a possible mechanism was proposed in photocatalytic reaction.

## Data Availability Statement

The datasets generated for this study are available on request to the corresponding author.

## Author Contributions

JW performed experiments and drafted the manuscript. YX provided the idea and guided the undertaken of the experiments. YL helped to discuss some of the experimental results. YD took the instrumental analysis and analyzed the data. JL and SL discussed the mechanisms of the photocatalysis reaction. JZ polished the language of the manuscript.

### Conflict of Interest

The authors declare that the research was conducted in the absence of any commercial or financial relationships that could be construed as a potential conflict of interest.
